# Integrated multi-omics identifies macrophage ARG1-mediated deacetylation with causal and diagnostic implications in ischemic stroke

**DOI:** 10.1016/j.isci.2026.115269

**Published:** 2026-03-06

**Authors:** Hang-Ze Ruan, Ying Zhu, Shen-chi Cheng, Jin-Yu Huang, Wei Hu

**Affiliations:** 1 Department of Critical Care Medicine, Affiliated Hangzhou First People’s Hospital, School of Medicine, Westlake University, Hangzhou, Zhejiang, China; 2 School of Medicine, Zhejiang University, Hangzhou, Zhejiang, China; 3 Department of Cardiology, Affiliated Hangzhou First People’s Hospital, School of Medicine, Westlake University, Hangzhou, Zhejiang, China; 4 The Fourth School of Clinical Medicine, Zhejiang Chinese Medical University, Hangzhou First People’s Hospital, Hangzhou, Zhejiang, China

**Keywords:** Health sciences, Medicine, Medical specialty, Health informatics, Internal medicine, Cardiovascular medicine

## Abstract

Ischemic stroke (IS) involves transcriptional dysregulation linked to epigenetic deacetylation. By integrating multi-omics data from IS patients and an MCAO model, we identified fifty-two differentially expressed deacetylation-related genes (DEARGs) and derived a three-gene signature (*VIM*, *ARG1*, and *PRPF31*) that exhibited high diagnostic accuracy (*AUC* = 0.840) and stratified IS patients into two immunologically distinct molecular subtypes with divergent immune microenvironment infiltration patterns. Two-sample Mendelian randomization analysis with 24 eQTL-derived instrumental variables confirmed *ARG1* as a causal factor for IS (IVW method; FDR-corrected *p* = 0.03). Single-cell RNA sequencing validated specific *ARG1* upregulation in infiltrating macrophages of a mouse MCAO model and myeloid subsets of human IS PBMCs, with RT-qPCR further verifying this pattern. Together, our study links this deacetylation-associated signature to immune heterogeneity in IS, identifies *ARG1*-expressing myeloid cells as potential mediators of epigenetic immune dysregulation, and provides a framework for stratified therapeutics.

## Highlights

• A three-gene deacetylation signature (*VIM*, *ARG1*, and *PRPF31*) accurately diagnoses ischemic stroke
• Two immune-distinct molecular subtypes enable patient stratification for personalized therapy
• Macrophage *ARG1* mediates ischemic stroke, linking deacetylation to immune dysregulation

## Graphical abstract

**Figure:**
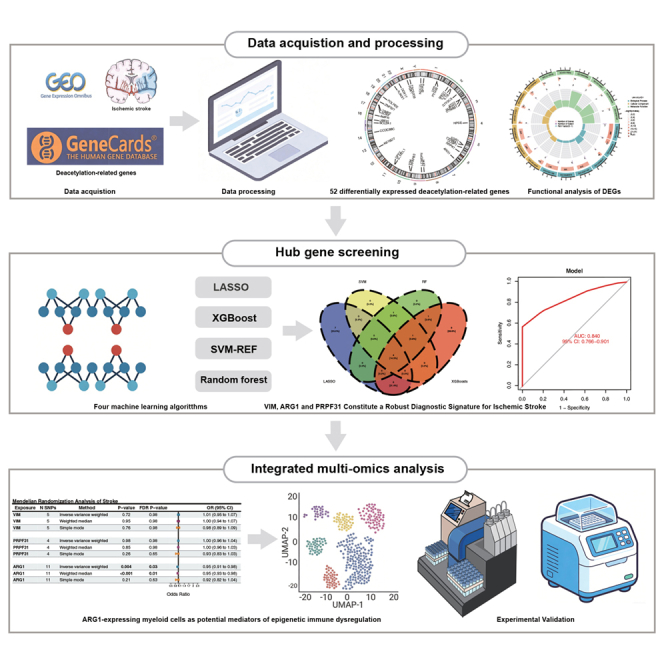

## Introduction

Ischemic stroke (IS), resulting from the sudden occlusion of a cerebral artery, is a leading cause of global mortality and chronic disability.^1^^,^^2^^,^^3^ The initial ischemic insult triggers a complex pathological cascade—including excitotoxicity, oxidative stress, and robust neuroinflammation—that amplifies secondary brain injury.^4^^,^^5^^,^^6^^,^^7^ While these core mechanisms are well-recognized, pronounced heterogeneity in etiology, clinical presentation, and treatment response among IS patients underscores the urgent need for improved molecular stratification and personalized therapeutic strategies.

Advances in high-throughput genomics have begun to elucidate the transcriptional architecture of IS.^8^^,^^9^ Bulk transcriptomic studies have identified numerous differentially expressed genes and pathways, yet they inherently obscure cell-type-specific dynamics. The emergence of single-cell RNA sequencing (scRNA-seq) now permits resolution of cellular heterogeneity within the neurovascular unit.^10^^,^^11^ However, an integrative framework that links bulk tissue findings with single-cell insights to dissect the role of specific regulatory gene families—such as those governing protein deacetylation—is still lacking. Deacetylation, a key reversible post-translational modification mediated by deacetylases, regulates broad biological processes—from chromatin remodeling and transcription factor activity to metabolic signaling—by removing acetyl groups from histone and non-histone proteins.^12^^,^^13^^,^^14^ Although certain deacetylases have been implicated in neuronal survival and inflammatory responses, a systematic assessment of the collective role of deacetylation-related genes in IS pathogenesis, and their potential to define molecular subtypes, remains unexplored.^15^^,^^16^^,^^17^^,^^18^ Moreover, establishing a causal—rather than correlative—link between deacetylation-related genes expression and IS risk is essential to overcome limitations inherent in observational transcriptomic studies.

To address these gaps, we performed an integrated multi-omics analysis to characterize the expression patterns, diagnostic potential, and functional significance of deacetylation-related genes in IS. We hypothesized that a distinct set of deacetylation-related genes drives IS pathogenesis and associates with specific immune microenvironment features. Using differential expression analysis and machine learning, we derived a diagnostic deacetylation-related genes signature. Consensus clustering based on deacetylation-related genes expression defined patient subtypes, which were characterized by distinct immune infiltration patterns. We further applied two-sample Mendelian randomization to infer causal relationships and integrated cross-species scRNA-seq data to pinpoint cellular sources of key genes and map the remodeled immune landscape. Key findings were validated in an experimental middle cerebral artery occlusion (MCAO) model. Together, this work highlights a deacetylation-centered perspective for understanding IS heterogeneity and informs potential biomarkers and therapeutic targets in IS.

## Results

### Deacetylation-related genes show dysregulated expression in IS

**Figure 1:**
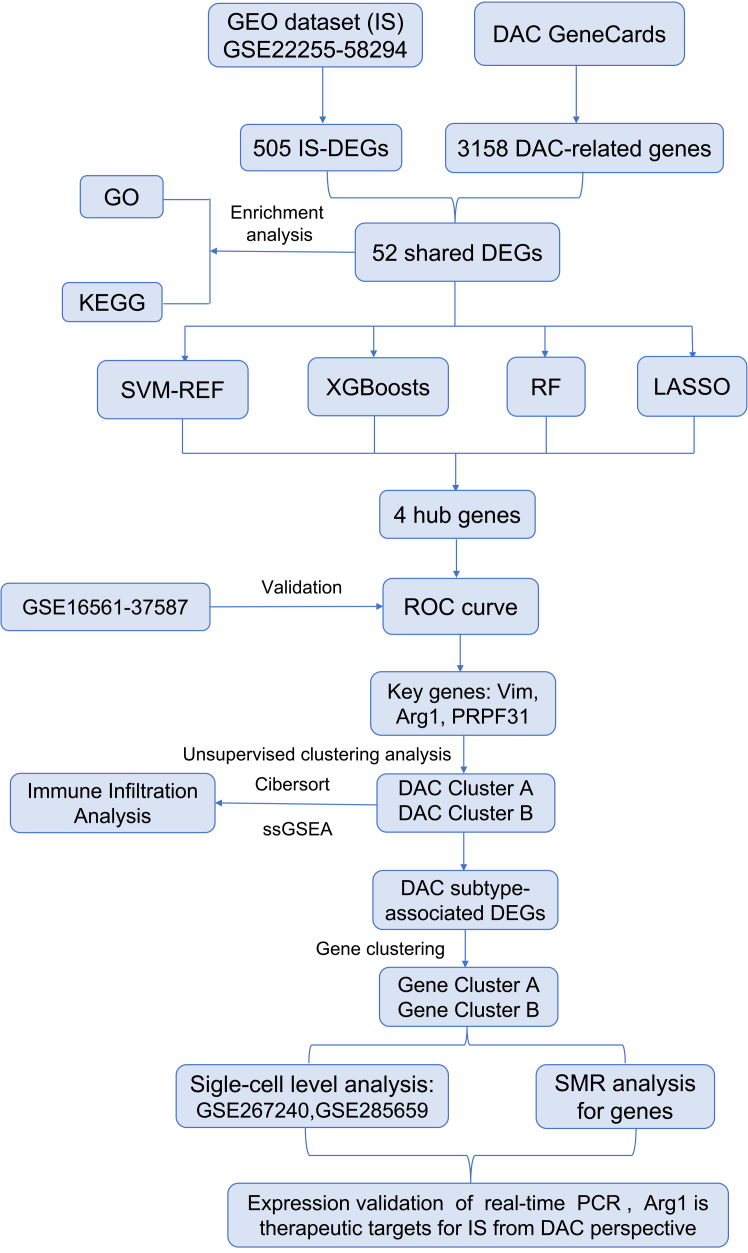
Bioinformatic screening identifies treatment-responsive deacetylation-related molecules in ischemic stroke Workflow of multi-step bioinformatic screening for deacetylation-related treatment-responsive molecules in ischemic stroke (IS) samples.

**Figure 2:**
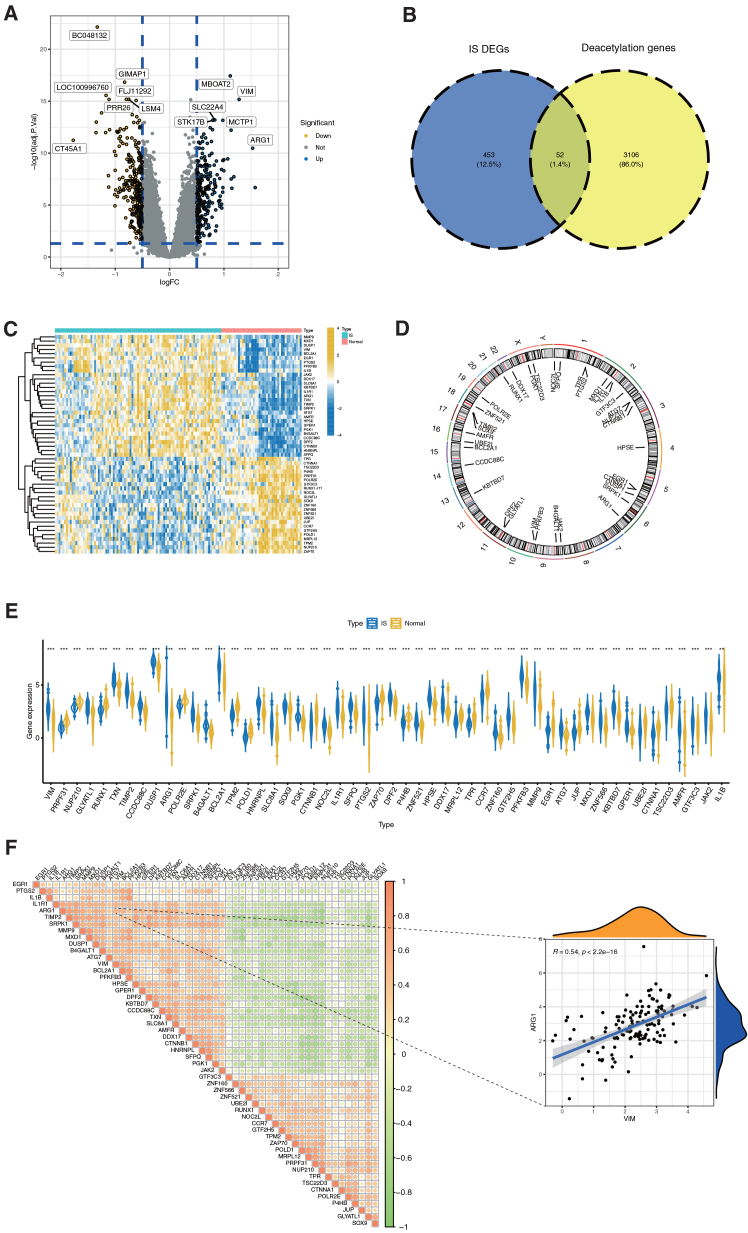
Deacetylation-related genes are differentially expressed in human IS blood samples (A) Volcano plot depicting differentially expressed genes (DEGs) between normal and IS samples. (B) Venn diagram identifying the intersection between IS-related DEGs and deacetylation-related genes. (C) Heatmap displaying the expression patterns of the 52 intersected deacetylation-related DEGs. (D) Circos plot illustrating the chromosomal locations of the 52 key deacetylation-related DEGs. (E) Violin plot depicting the 52 differentially expressed deacetylation-related genes in IS. (F) Correlation matrix of the 52 differentially expressed deacetylation-related genes . ∗*p* < 0.05, ∗∗*p* < 0.01, ∗∗∗*p* < 0.001 by Wilcoxon rank-sum test.

The study design is summarized in Figure 1. A volcano plot revealed 505 differentially expressed genes (DEGs) between normal and IS samples (Figure 2A). Among these, 52 genes overlapped with a set of 3,158 deacetylation-related genes and were defined as differentially expressed deacetylation-related genes (DEARGs) (Figure 2B). A heatmap visualization showed distinct expression patterns of these 52 DEARGs, with some upregulated and others downregulated in IS samples (Figures 2C and E). Figure 2D depicts the chromosomal distribution of the 52 DEARGs, clearly demonstrating that they are scattered across multiple chromosomes rather than concentrated in a few specific regions—indicating a non-random yet widely dispersed genomic distribution pattern. Correlation analysis further indicated a strong positive association between *ARG1* and *VIM* (Figure 2F).

### DEARGs are enriched in immune and cardiovascular pathways in IS

**Figure 3:**
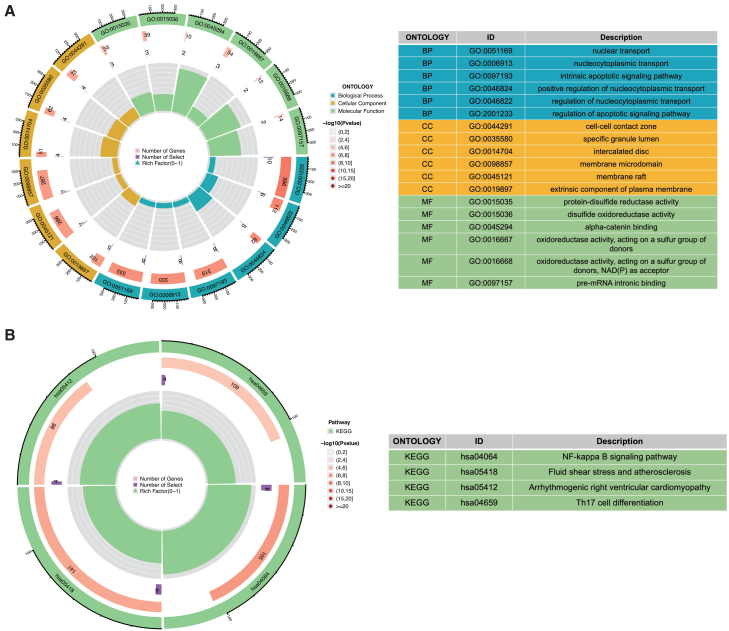
Differentially expressed deacetylation-related genes enrich in IS-associated biological functions and pathways (A) Chord diagram of GO enrichment results (BP/CC/MF) for deacetylation-related DEGs; the right table lists enriched GO terms with ontology category, ID and functional description. (B) KEGG pathway enrichment analysis for deacetylation-related DEGs; the right table details enriched KEGG pathways with ID and description.

Functional enrichment analysis of the 52 DEARGs elucidated their potential roles in IS. GO terms were significantly enriched in biological processes including protein acetylation, cellular components such as the cell-cell contact zone, membrane microdomain, and membrane raft, and molecular functions including disulfide oxidoreductase activity (Figure 3A). KEGG pathway analysis further showed enrichment in Th17 cell differentiation, the NF-κB signaling pathway, fluid shear stress and atherosclerosis, and arrhythmogenic right ventricular cardiomyopathy (Figure 3B). DO enrichment analysis showed that the 52 DEARGs were associated with hepatic vascular disease, systemic hypertension, and cardiomyopathy (Figures S1A and S1B). These findings collectively implicate the DEARGs in intercellular signaling processes relevant to both neuronal and cardiovascular pathophysiology in IS.

### *VIM*, *ARG1*, and *PRPF31* form a robust diagnostic signature for IS

**Figure 4:**
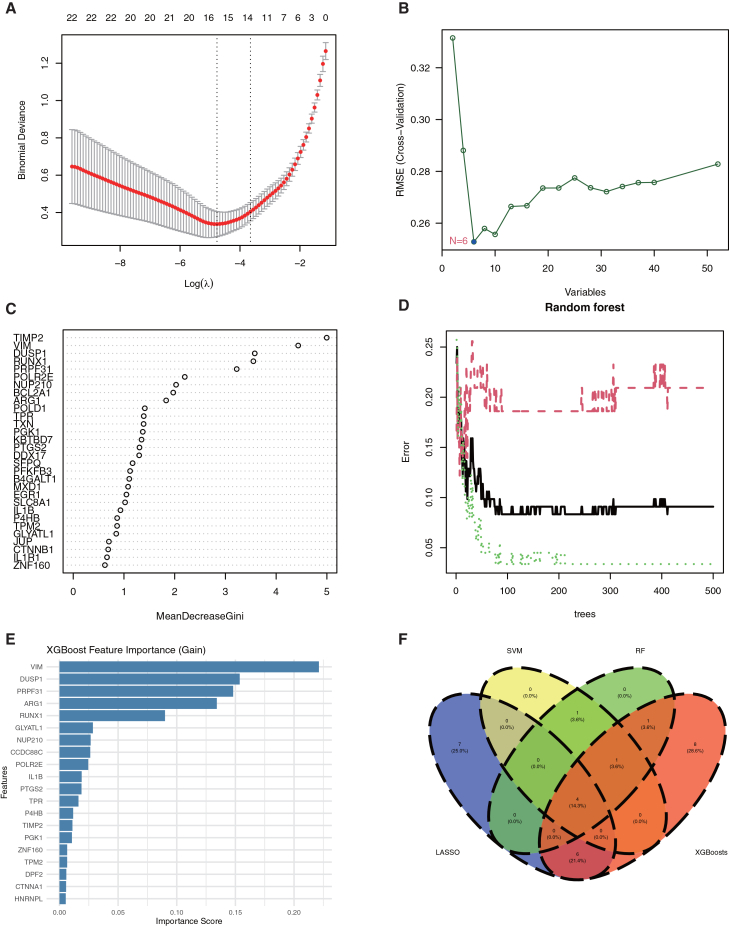
Multi-machine learning strategies identify four core deacetylation-related genes for IS prognostic signature construction (A) Lasso coefficient profiles plotted against the log(λ) values. The vertical dashed line indicates the optimal λ value (lambda.min) selected by 10-fold cross-validation, which resulted in the retention of seven genes. (B) Root-mean-square error (RMSE) values from 15-fold cross-validation using SVM-RFE. The point corresponding to the minimal RMSE, which represents the optimal gene subset identified by SVM, is highlighted. (C) Variable importance plot based on mean decrease accuracy (MDA) from the random forest model. Genes are ranked according to their contribution to predictive accuracy. (D) Out-of-bag (OOB) error rate of the random forest model as a function of the number of decision trees. The error stabilizes as the number of trees increases, indicating model convergence. (E) Feature importance scores derived from SHapley Additive exPlanations (SHAP) analysis, representing the average marginal contribution of each feature across all possible subsets. (F) Venn diagram illustrating the overlap of IS-associated genes identified by the four distinct feature selection methods: Lasso regression, SHAP analysis, random forest, and SVM-RFE.

**Figure 5:**
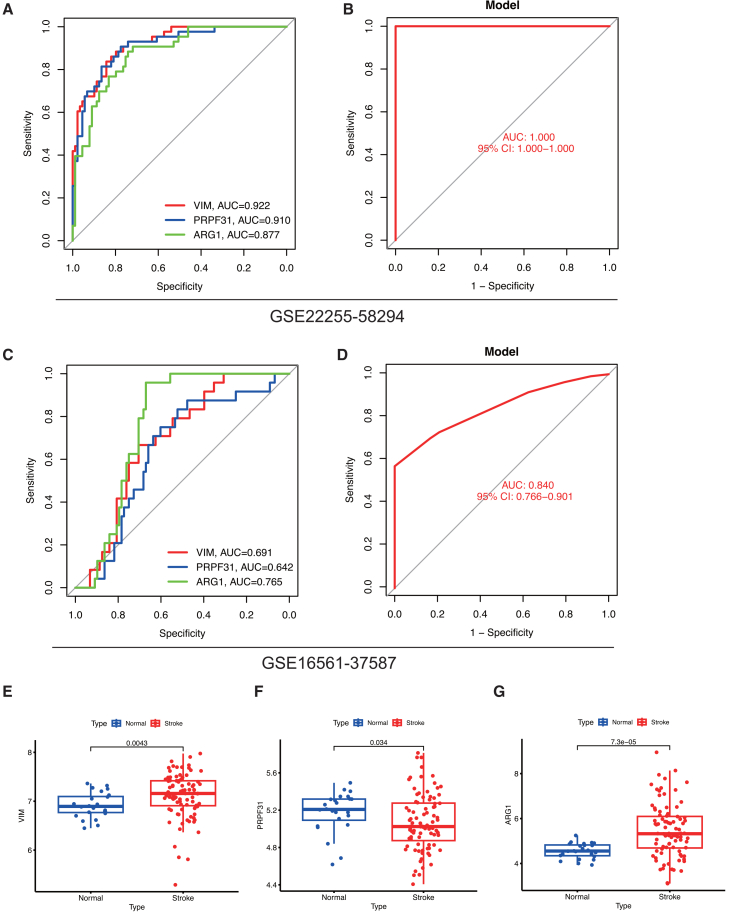
The *VIM-PRPF31-ARG1* three-gene signature exhibits high diagnostic accuracy for IS (A and B) Receiver operating characteristic (ROC) curves demonstrating the discriminatory power of the three-gene signature in the GSE22255-58294 training cohort. The area under the curve (AUC) values indicates high diagnostic accuracy. (C and D) ROC curves validating the diagnostic efficacy of the three-gene signature in the independent validation cohort GSE16561-37587. (E, F, and G) Validation of differential expression patterns of *VIM* (E), *PRPF31* (F)*,* and *ARG1* (G) between normal controls and ischemic stroke samples in the GSE16561-37587 dataset. ∗*p* < 0.05, ∗∗*p* < 0.01 by Wilcoxon rank-sum test.

To identify key deacetylation-related predictors of IS, we applied multiple machine learning algorithms. LASSO regression and SVM selected seven and six feature genes, respectively (Figures 4A and 4B). Using 500 trees in the RF model ensured error stability (Figures 4C and 4D). RF feature importance ranking highlighted *TIMP2*, *VIM*, and *DUSP1* as top contributors, a finding supported by SHAP analysis, which confirmed *VIM* as the most influential feature (Figure 4E). Integration of results from all four methods identified four core genes: *RUNX1*, *VIM*, *ARG1*, and *PRPF31* (Figure 4F). We further evaluated the diagnostic potential of these genes using ROC analysis and validated the results in the GSE16561-37587 dataset (Figure 5). Individually, the three core genes effectively distinguished IS from controls, with *AUCs* of 0.922 for *VIM*, 0.877 for *ARG1*, and 0.91 for *PRPF31* (Figure 5A). A combined model achieved perfect separation in the training set (*AUC* = 1.000; Figure 5B). This performance was maintained in the independent validation set, where the model yielded an *AUC* of 0.840 (Figure 5D), with individual *AUCs* of 0.691 (*VIM*), 0.765 (*ARG1*), and 0.642 (*PRPF31*) (Figure 5C). Expression validation confirmed upregulation of *VIM* and *ARG1* and downregulation of *PRPF31* in IS samples (Figures 5E–5G), supporting the robustness of this three-gene signature for IS diagnosis. In contrast, *RUNX1* exhibited limited diagnostic utility in our evaluation: its *AUC* of 0.531 approached 0.5 (the threshold for random classification), and this, coupled with the absence of statistically significant differences in *RUNX1* expression between IS and normal groups, indicates *RUNX1* lacks meaningful disease-associated expression alterations and cannot serve as a reliable diagnostic biomarker for distinguishing IS from normal status (Figures S2A and S2B).

### IS patients stratify into two DEARG-associated subtypes with immune-related transcriptomic features

**Figure 6:**
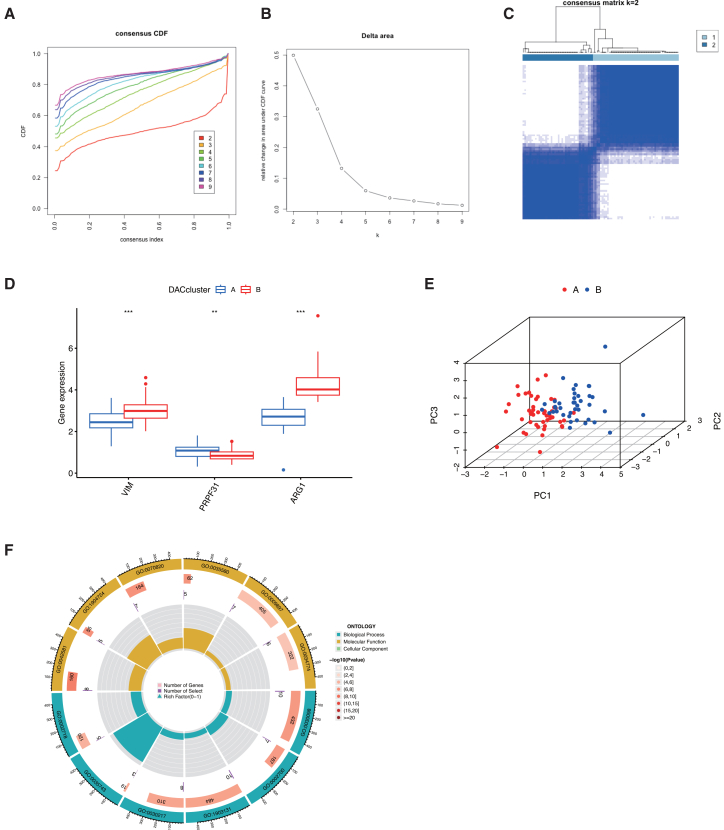
Consensus clustering of three deacetylation-related genes classifies IS into two distinct molecular subtypes (A) Cumulative distribution function (CDF) curves for consensus clustering solutions across k values ranging from 2 to 9. (B) Relative change in the area under the CDF curve for consecutive k values, used to determine the optimal cluster number. (C) Consensus matrix heatmap for the optimal cluster solution (k = 2), demonstrating high consensus within clusters and low consensus between clusters. (D) Differential expression patterns of the three deacetylation-related genes between the two identified subtypes; ∗∗∗*p* < 0.001, ∗∗*p* < 0.01, ∗*p* < 0.05 by Wilcoxon rank-sum test. (E) Principal-component analysis (PCA) plot showing distinct transcriptomic profiles between subtype A and subtype B. (F) GO enrichment analysis of biological processes significantly associated with the hub genes in the identified ischemic stroke subtypes. The most significantly enriched terms are shown (*p* < 0.05).

Based on the expression of three key deacetylation-related DEGs, we performed consensus clustering to molecularly subtype IS patients. With the cluster number (k) set from 1 to 9, k = 2 was identified as optimal, dividing the 89 IS samples into two distinct subtypes (A and B) (Figures 6A–6C). A histogram confirmed differential expression of the three genes between the subtypes (Figure 6D), and principal component analysis showed clear separation between them (Figure 6E). Subsequently, we identified 112 deacetylation-related DEGs across these two subtypes. GO enrichment analysis of these genes revealed significant involvement in immune-related processes, including positive regulation of B cell-mediated immunity, monocyte differentiation, CD8^+^ alpha-beta T cell homeostasis, and T cell differentiation (Figure 6F). These results imply that deacetylation-related genes play a central role in the immunopathology of IS by modulating the differentiation and function of multiple immune cells, such as monocytes, T cells, and B cells.

### DEARG-associated subtypes exhibit distinct immune infiltration patterns in Ischemic Stroke

**Figure 7:**
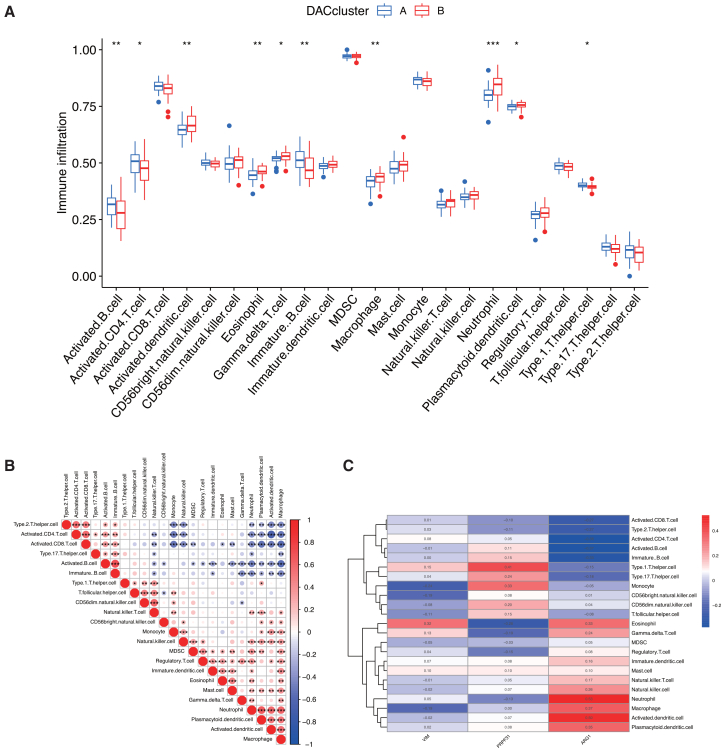
Key candidate biomarkers modulate immune cell infiltration during post-stroke recovery (A) Comparison of immune cell infiltration abundances between DAC subtypes. (B) Correlation network depicting significant interactions among immune cells by ssGSEA. (C) Correlation heatmap depicting associations of *VIM*, *ARG1*, and *PRPF31* with infiltrating immune infiltration cells. ∗∗∗*p* < 0.001, ∗∗*p* < 0.01, ∗*p* < 0.05 by Wilcoxon rank-sum test.

To characterize the immune landscape underlying the two deacetylation-related subtypes, we compared immune cell infiltration between Cluster A and Cluster B using ssGSEA. Cluster A exhibited significantly higher levels of activated B cells, activated CD4^+^ T cells, immature B cells, and type-1 T helper cells. In contrast, cluster B showed markedly greater infiltration of activated dendritic cells, eosinophils, gamma delta T cells, macrophages, neutrophils, and plasmacytoid dendritic cells (Figure 7A). Correlation analysis across immune cell types revealed a strong positive association between immature and activated B cells, and a pronounced negative correlation between activated dendritic cells and activated CD4^+^ T cells (Figure 7B). To further explore the relationship between the deacetylation modification and the immune microenvironment of IS, the infiltration levels of key immune subsets were significantly associated with the expression of the three central deacetylation-related genes (Figure 7C), underscoring a potential regulatory link between deacetylation and the immune microenvironment in IS.

To validate whether these subtype-specific immune features are reflected in the overall IS population, we further performed CIBERSORTx deconvolution on bulk RNA-seq data (GSE199819). Consistent with the subtype-level observations, this independent analysis indicated a significant increase in the abundances of memory B cells, plasma cells, and CD8^+^ T cells in IS samples compared to controls (Figure S3). Collectively, these results confirm the coordinated activation of both cellular immunity (CD4^+^ T cells, CD8^+^ T cells, and γδ T cells) and humoral immunity (B cell subsets, plasma cells) during the pathological progression of IS, while highlighting that deacetylation modification may act as a key regulator of immune microenvironment heterogeneity in IS.

### Two deacetylation gene patterns correlate with DEARG subtypes and deacetylation scores

**Figure 8:**
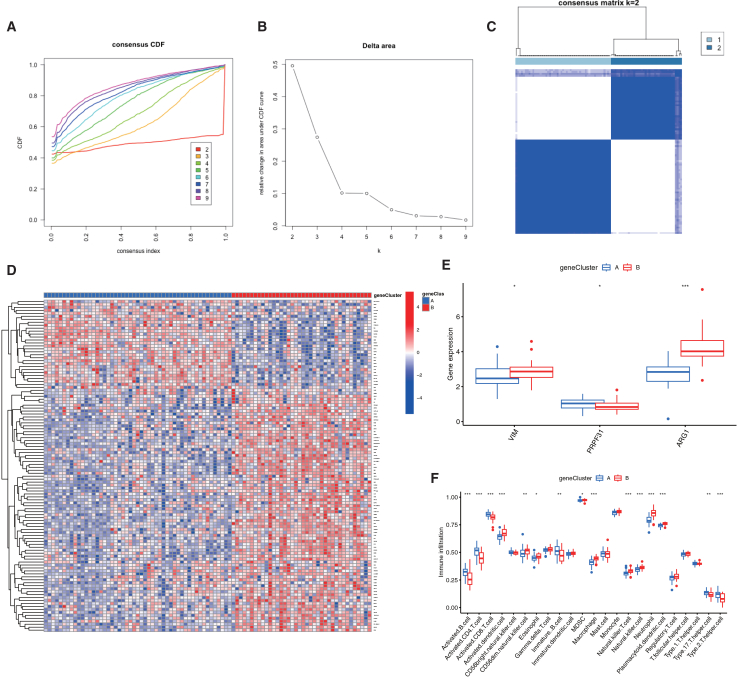
Molecular subtyping of ischemic stroke via consensus clustering of 112 deacetylation-related DEGs (A) Cumulative distribution function (CDF) curves for consensus clustering at k values ranging from 2 to 9. (B) Relative change in the area under the CDF curve for consecutive k values. (C) Consensus matrix illustrating cluster stability for the optimal k = 2. (D) Expression heatmap of the 112 deacetylation-related DEGs across the two identified gene clusters (cluster A and cluster B). (E) Differential expression analysis of three representative deacetylation-related DEGs between gene cluster A and cluster B. (F) Comparison of immune cell infiltration levels between the two gene clusters. ∗∗∗*p* < 0.001, ∗∗*p* < 0.01, ∗*p* < 0.05 by Wilcoxon rank-sum test.

**Figure 9:**
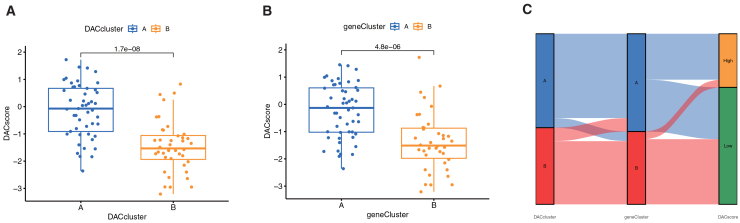
Deacetylation scores effectively discriminate IS molecular subtypes and patient stratification (A and B) Comparison of deacetylation scores between (A) DAC clusters A and B, and (B) gene expression clusters A and B. (C) Sankey diagram showing the correspondence among DAC-based clusters, gene expression clusters, and deacetylation score groups; ∗∗∗*p* < 0.001, ∗∗*p* < 0.01, ∗*p* < 0.05 by Wilcoxon rank-sum test.

To further validate the two deacetylation patterns, we performed consensus clustering of IS patients using the 112 deacetylation-related DEGs, which defined two distinct genomic subtypes—gene cluster A and gene cluster B—consistent with the earlier patterns (Figures 8A–8C). Expression patterns of the 112 DEGs across clusters are shown in a heatmap (Figure 8D), and the three central deacetylation-related genes displayed marked expression differences between clusters (Figure 8E). Immune infiltration profiles also aligned with the deacetylation subtypes, reinforcing the classification robustness (Figure 8F). Finally, deacetylation scores calculated from PCA were significantly elevated in both subtype A and gene cluster A compared to their counterparts (Figures 9A and 9B). A Sankey diagram illustrated the correspondence among deacetylation patterns, gene clusters, and deacetylation scores (Figure 9C).

### *ARG1* exerts a causal protective effect against IS risk

**Figure 10:**
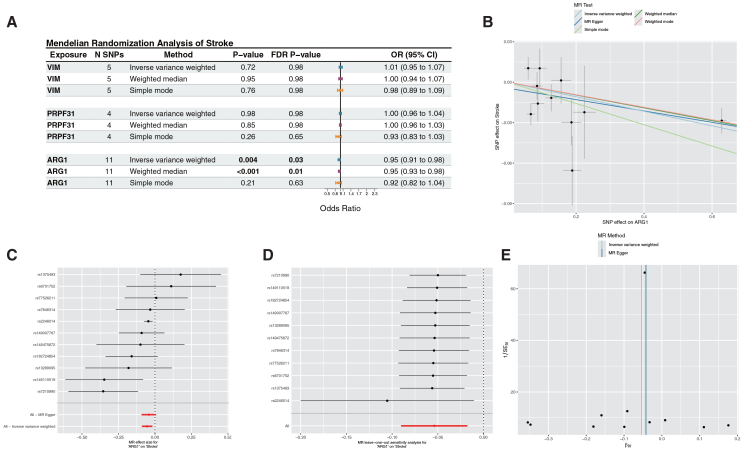
Mendelian randomization identifies *ARG1* as a causal protective factor for IS (A) Forest plot of MR model results. (B) Scatterplot of the five MR models. Each point represents an IV, with the line on each point indicating the 95% CI. The y axis shows the effect of SNPs on the outcome, and the x axis shows the effect of SNPs on exposure. (C) Forest plot of MR analysis results for each *ARG1*-associated SNP; the red line represents the pooled effect of all SNPs, indicating that increased *ARG1* expression is associated with reduced ischemic stroke risk. (D) MR sensitivity analysis for *ARG1* after removing SNPs using the leave-one-out method. The red line represents the pooled results for all SNPs. (E) Funnel plot of 11 SNPs on MR analysis.

Expression quantitative trait locus mapping is a well-established approach for elucidating the influence of common genetic variants on interindividual variation in gene expression. To investigate the role of such variants in IS, we identified 24 eQTLs associated with the expression of three target markers after clustering SNPs in linkage disequilibrium (r^2^ < 0.001). The average F-statistic for the SNPs used as instrumental variables ranged from 21.30 to 1884.49, indicating that they were robust instruments. Using publicly available GWAS data, we performed a two-sample MR analysis in which eQTL SNPs served as instrumental variables, the markers as exposures, and IS as the outcome. The MR analysis demonstrated a direct causal relationship between *ARG1* and IS risk. Specifically, *ARG1* (IVW method, 11 SNPs, OR = 0.95, 95% CI = 0.93–0.98, raw *p* = 0.004, FDR-corrected *p* = 0.03) was identified as a protective factor for stroke, whereas *VIM* (IVW, 5 SNPs, raw *p* = 0.72, FDR-corrected *p* = 0.98) and *PRPF31* (IVW, 4 SNPs, raw *p* = 0.98, FDR-corrected *p* = 0.98) showed no association with stroke pathogenesis (Figure 10A). Sensitivity analyses—including scatter, forest, funnel, and leave-one-out plots (Figures 10B–10E)—further supported a protective role for *ARG1*.

### *Arg1* is specifically upregulated in macrophages in a mouse IS model

**Figure 11:**
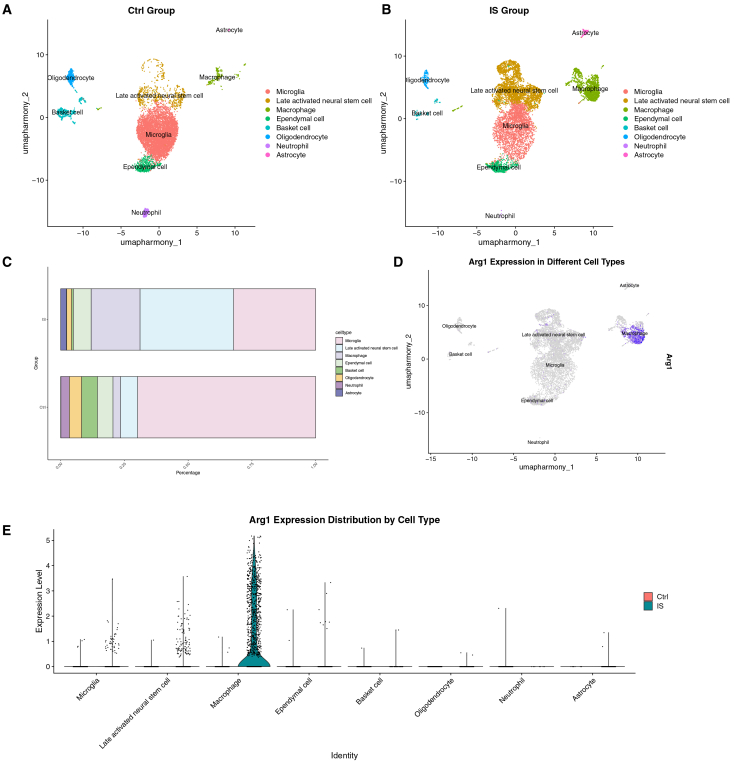
Single-cell RNA-seq reveals *Arg1* is specifically expressed in mouse IS brain cell subsets (A and B) The cellular distribution of 8 cell clusters between sham and MCAO mice. (C)The bar plot shows the cell ratio between ischemic stroke mice and sham mice. (D) The cellular distribution of *Arg1* in MCAO mice. (E) The cellular distribution of *Arg1* between sham and MCAO mice.

To characterize cellular heterogeneity in IS, we performed single-cell RNA sequencing on dataset GSE267240, which comprises data from two IS and two sham-operated mice (used due to the unavailability of human scRNA-seq data). UMAP visualization revealed eight distinct cell clusters: astrocytes, late-activated neural stem cells, ependymal cells, basket cells, neutrophils, macrophages, microglia, and oligodendrocytes (Figures 11A and 11B). Comparative analysis showed a significant increase in the proportions of macrophages and late-activated neural stem cells in the stroke group, whereas microglia were markedly reduced (Figures 11C and 11D). Notably, *Arg1* expression was significantly elevated in macrophages (Figure 11E), which is consistent with rodent IS models reported in the literature.^19^ Integrated analysis of both bulk transcriptome and single-cell RNA-seq data identified macrophages as key cellular mediators in IS.

### *ARG1* upregulation in myeloid cells drives PBMC immune remodeling in IS patients

**Figure 12:**
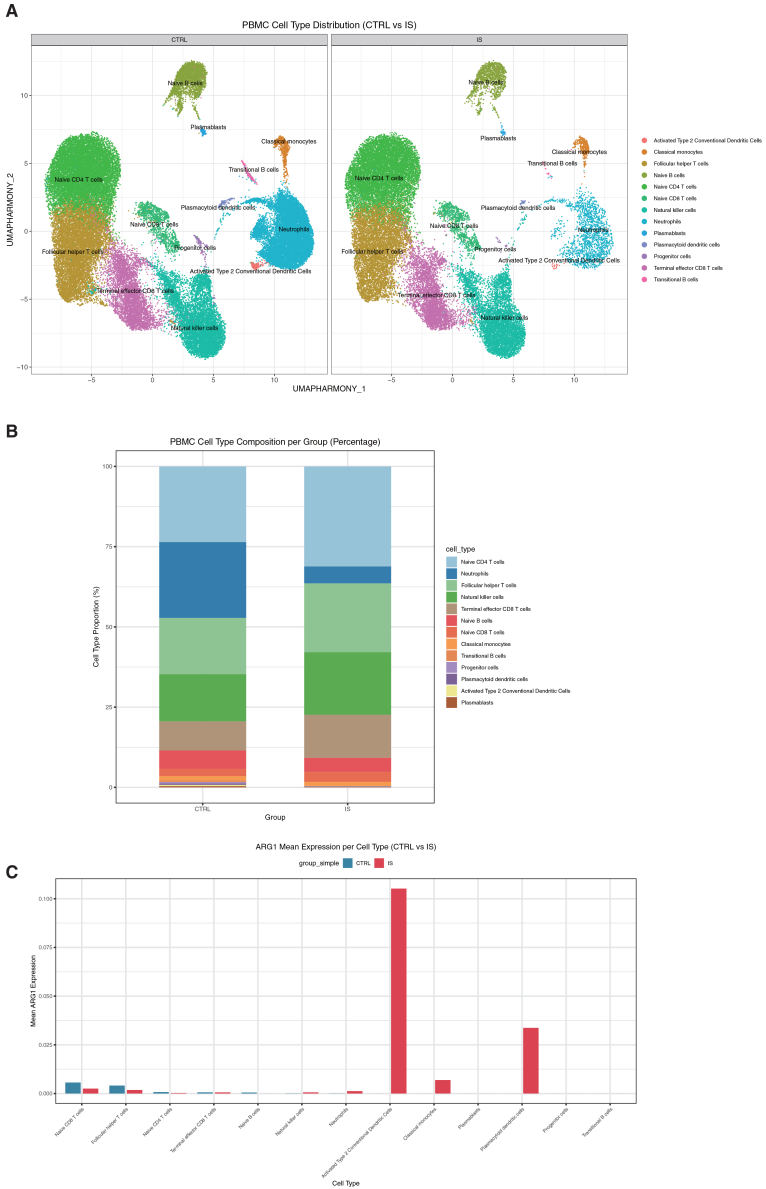
Single-cell RNA-seq of human PBMCs identifies *ARG1* expression in IS patient immune subsets (A) Cell clusters were identified with UMAP projection of 90751 cells from controls and patients who had a stroke. (B) Bar plot of PBMC subset proportion between controls and IS patients. (C) Mean expression level of *ARG1* in PBMC subtypes.

To clarify the role of *ARG1* in the immune microenvironment of IS, we investigated the specific immune cell populations driving *ARG1* elevation by performing analyses on a scRNA-seq dataset (GSE285659) of peripheral blood mononuclear cells (PBMCs). After quality control, we analyzed 56,242 genes from 34,583 cells of patients who had a stroke (*n* = 3), and 59,115 genes from 56,168 cells of controls (*n* = 3). Using graph-based clustering of UMAP, we identified 13 cell clusters (Figure 12A) in each subject based on the expression of canonical marker genes. Identified cell clusters and marker genes are naive CD4^+^ T cells clusters (TCF7 and CCR7), follicular helper T cells (CXCR5 and PDCD1), neutrophils (S100A8 and S100A12), natural killer cells (GNLY), terminal effector CD8^+^ T cells (CCL5 and CD8A), naive B cells (CD79A and MS4A1), naive CD8^+^ T cells (CD8A) and classical monocytes (C1QA and SIGLEC10), transitional B cells (IGHD), plasmacytoid dendritic cells (CLEC4C and LILRA4), progenitor cells (PROM1), plasmablasts (JCHAIN and IGHA1), and activated type 2 conventional dendritic cells (CLEC10A). Comparative analysis revealed remodeling of the PBMC immune landscape in IS patients, with increased proportions of naive CD4^+^ T cells, follicular helper T cells, and terminal effector CD8^+^ T cells alongside decreased neutrophils (Figure 12B); notably, cell-type-specific *ARG1* upregulation was detected in three myeloid subsets—plasmacytoid dendritic cells, activated type 2 conventional dendritic cells, and classical monocytes (Figures 12C and S4)—indicating that these compositional shifts represent the cellular basis for elevated *ARG1* activity post-stroke. To further validate these findings and explore the functional consequences of *ARG1* upregulation, we analyzed immune cell infiltration differences between patients with high and low *ARG1* expression, which showed that the *ARG1*-high group exhibited greater infiltration of activated dendritic cells, eosinophils, γδ T cells, macrophages, and neutrophils, but reduced infiltration of activated B cells, activated CD4^+^/CD8^+^ T cells, immature B cells, and type 1/2 helper T cells (Th1/Th2 cells) compared to the *ARG1*-low group (Figure S5). Collectively, these results demonstrate that high *ARG1* expression modulates immune cell infiltration, skewing the immune microenvironment toward inflammation/innate immunity while suppressing adaptive cytotoxic immunity—one of the key mechanisms by which *ARG1* influences immune responses and prognosis in IS.

### RT-qPCR validates elevated *Arg1* expression in an MCAO model

**Figure 13:**
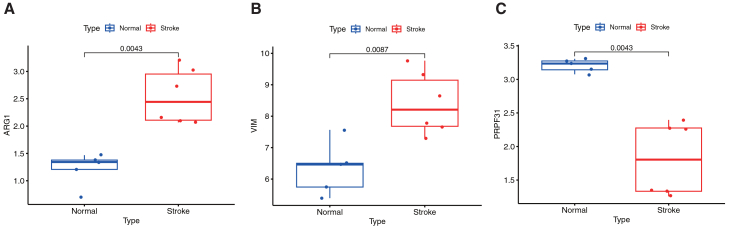
Relative mRNA expression levels of *Arg1*, *Vim*, and *Prpf31*: Comparison between MCAO rats and healthy control rats (A–C) Relative mRNA expression levels of *Arg1* (A), *Vim* (B), and *Prpf31* (C) in MCAO rats vs. healthy control rats. ∗∗∗*p* < 0.001, ∗∗*p* < 0.01, ∗*p* < 0.05 by Wilcoxon rank-sum test.

To validate candidate genes from GEO analysis, we measured their expression by RT-qPCR in an MCAO model. All samples had RNA concentrations within the acceptable range. The MCAO group showed significantly increased *Arg1* mRNA levels compared to controls (*p* < 0.05; Figures 13A–13C), consistent with the bioinformatic predictions from the GSE22255-58294 datasets.

## Discussion

IS remains a leading cause of global mortality and long-term disability, with its pathophysiology involving intricate crosstalk among neuroinflammation, vascular dysfunction, and metabolic disturbance.^2^^,^^20^^,^^21^ Despite advances in acute intervention, the absence of reliable molecular biomarkers for early diagnosis and subtype-specific therapeutic targets continues to limit clinical management. Protein deacetylation—an essential epigenetic mechanism regulated by deacetylases—modulates diverse cellular processes including apoptosis, immune activation, and vascular homeostasis,^15^ yet the systematic expression profiles, causal relevance, immune associations, and clinical utility of DEARGs in IS remain incompletely elucidated. To address this gap, we employed an integrated multi-omics approach combined with multidimensional analyses to decipher the deacetylation-related gene landscape in IS, identifying 52 dysregulated DEARGs enriched in IS-relevant pathways (Th17 cell differentiation, NF-κB signaling), a robust three-gene signature (*VIM*, *ARG1*, and *PRPF31*) with high diagnostic efficacy (*AUC* = 1.000 in training set, *AUC* = 0.840 in validation), two distinct deacetylation-related molecular subtypes with divergent immune profiles, and *ARG1* as a direct causal protective factor for IS (IVW method; FDR-corrected *p* = 0.03) via MR—with *ARG1* upregulation being cell-type-specific and underpinning post-stroke immune remodeling. Collectively, our findings demonstrate profound deacetylation dysregulation in IS linked to its molecular subtypes, immune landscape, and causal pathogenesis, establishing *ARG1* as a central driver of immune dysregulation and a promising therapeutic target while providing a framework for precision diagnosis and personalized treatment to advance IS pathophysiology research and clinical translation.

### Expression landscape and functional relevance of differentially expressed DEARGs

Through comparative transcriptomic analysis, we identified 505 DEGs in IS, including 52 DEARGs that demonstrate distinct expression patterns and specific chromosomal distributions (Figures 2A–2E). Functional enrichment analysis revealed these DEARGs are significantly involved in protein acetylation processes, membrane microdomain organization, and disulfide oxidoreductase activity (Figure 3A). These molecular functions collectively contribute to IS pathogenesis through several mechanisms: protein acetylation regulates neuronal survival and blood-brain barrier integrity, while membrane microdomains facilitate critical signaling complex assembly.^22^^,^^23^^,^^24^ Further pathway analysis identified enrichment in Th17 cell differentiation, NF-κB signaling, and fluid shear stress responses (Figure 3B), with NF-κB activation driving neuroinflammatory cascades and Th17 cells amplifying blood-brain barrier disruption through IL-17 secretion.^25^^,^^26^^,^^27^ Notably, the scattered distribution of DEARGs across multiple chromosomes (rather than clustering in focal chromosomal regions) excludes the possibility that their differential expression is driven by localized genomic abnormalities (Figure 2D). Instead, this distribution pattern indicates that the dysregulation of deacetylation-related genes in IS is a systemic, genome-wide regulatory response—one that aligns closely with our functional enrichment results, as DEARGs collectively participate in diverse IS-relevant pathways. This convergence of genomic localization and functional data emphasizes that deacetylation modification does not rely on isolated genetic alterations but involves the coordinated regulation of multiple genomic loci, reflecting the complexity of its role in IS pathophysiology.

Moreover, the involvement of the fluid shear stress and atherosclerosis pathway reveals the potential role of DEARGs in IS-related vascular lesions (atherosclerotic progression). Additionally, disease ontology enrichment analysis demonstrated that these DEARGs are associated with hepatic vascular disease, systemic hypertension, and cardiomyopathy—common comorbidities or risk factors of IS, highlighting their regulatory value in the cross-pathophysiology of the nervous and cardiovascular systems. The convergence of DEARGs on these interconnected pathways emphasizes their coordinated regulation of inflammatory signaling, vascular pathology, and neuronal damage, thereby establishing deacetylation-mediated mechanisms as central contributors to IS progression.

### Identification of core deacetylation-related genes and their diagnostic potential

To identify key deacetylation-related regulators in IS, we applied an integrated machine learning approach. LASSO regression selected 7 feature genes, while SVM identified 6 genes (Figures 4A and 4B). Random Forest analysis with 500 trees (Figures 4C and 4D) and SHAP interpretation consistently highlighted *TIMP2*, *VIM*, and *DUSP1* as top contributors, with *VIM* emerging as the most influential predictor (Figure 4E). Integration across all methods yielded four core genes: *RUNX1*, *VIM*, *ARG1*, and *PRPF31* (Figure 4F). However, *RUNX1* was deemed not a reliable diagnostic biomarker for distinguishing IS from normal status due to its limited diagnostic utility, prompting us to subsequently focus on the three most diagnostically promising genes (*VIM*, *ARG1*, and *PRPF31*) for comprehensive evaluation. Receiver operating characteristic analysis demonstrated excellent diagnostic performance with individual *AUCs* of 0.922 (*VIM*), 0.877 (*ARG1*), and 0.91 (*PRPF31*) in the training set (Figure 5A). The combined three-gene signature achieved perfect discrimination (*AUC* = 1.000; Figure 5B) in training and maintained strong diagnostic accuracy (*AUC* = 0.840; Figure 5D) in the independent validation cohort, with individual validation *AUCs* of 0.691 (*VIM*), 0.765 (*ARG1*), and 0.642 (*PRPF31*) (Figure 5C). Expression validation confirmed significant upregulation of *VIM* and *ARG1* alongside downregulation of *PRPF31* in IS samples (Figures 5E–5G). The biological relevance of these markers supports their diagnostic value: *VIM* facilitates glial scar formation and promotes inflammatory cell infiltration following blood-brain barrier disruption^28^^,^^29^; *ARG1* depletes arginine to compromise nitric oxide-mediated vasodilation and exacerbate tissue hypoxia^30^^,^^31^; *PRPF31* regulates RNA splicing, with its downregulation potentially impairing cellular stress responses.^32^

Notably, beyond their individual diagnostic value, these three genes converge functionally onto a coherent pathological network centered on dysregulated protein deacetylation. As a core cytoskeletal protein, *VIM* serves as a direct target of histone deacetylases (HDAC1, HDAC6)^33^^,^^34^^,^^35^; alterations in its acetylation status are not only a direct consequence of deacetylase activity but also feedback to regulate cellular structure and signal transduction. *ARG1* exerts its effects at the metabolic level: by reprogramming arginine metabolism and energy sensing,^36^^,^^37^^,^^38^ it indirectly yet globally modulates the availability of acetyl-CoA and the activity of deacetylases,^39^^,^^40^ thereby providing the necessary metabolic milieu for deacetylation processes. In contrast, *PRPF31* represents a link at the level of gene expression regulation: as a core splicing factor, it ensures the correct processing of mRNA encoding numerous regulatory factors,^41^ and may include HDACs and histone acetyltransferases (HATs).^42^ Its dysfunction disrupts the “splicing-acetylation” coupling,^43^^,^^44^ thereby inducing widespread dysregulation of the deacetylation network. Thus, these three gene biomarkers, from three complementary dimensions (structural/signaling, metabolic, and post-transcriptional regulation), collectively point to and support the central role of aberrant protein (particularly histone) deacetylation in the initiation and progression of stroke. Our multi-omics data converge on this pathway, significantly enhancing the reliability of this biological interpretation. Additional validation in an MCAO model confirmed *ARG1* upregulation (Figures 13A–13C), consistent with bioinformatic predictions from GSE22255 and GSE58294 datasets. This multi-level validation establishes the three-gene signature as a promising diagnostic tool for IS.

### Deacetylation-associated subtypes and their immune landscape

To explore molecular heterogeneity in IS, we carried out consensus clustering using three core deacetylation-related genes. The optimal clustering configuration (k = 2) divided 89 IS samples into two molecular subtypes (A and B), which was corroborated by consensus matrix evaluation (Figures 6A–6C). The classification robustness was further supported by distinct expression patterns of the core genes across subtypes (Figure 6D) and clear separation in principal-component analysis (Figure 6E). Further analysis revealed 112 deacetylation-related differentially expressed genes between the two subtypes, showing significant enrichment in immune-related biological processes such as B cell-mediated immunity, monocyte differentiation, and T cell homeostasis and differentiation (Figure 6F). This suggests that deacetylation-related genes may help regulate immune cell function in IS pathogenesis.

Comprehensive immune characterization uncovered distinct microenvironmental patterns between the subtypes. Cluster A displayed an adaptive immune-dominant profile, marked by heightened infiltration of activated B cells, CD4^+^ T cells, immature B cells, and Th1 cells. In contrast, cluster B exhibited an innate immune-prevalent phenotype, with elevated levels of activated dendritic cells, eosinophils, γδ T cells, macrophages, neutrophils, and plasmacytoid dendritic cells (Figure 7A). Correlation analysis across immune cell types demonstrated coordinated immune responses, including a strong positive association between immature and activated B cells, and a notable negative correlation between activated dendritic cells and CD4^+^ T cells (Figure 7B). Importantly, expression levels of the core deacetylation-related genes were significantly correlated with specific immune cell subsets (Figure 7C), indicating potential functional crosstalk between deacetylation mechanisms and immune microenvironment composition. These molecular subtypes carry distinct therapeutic implications. The innate immunity-enriched cluster B may be more amenable to anti-inflammatory strategies that target macrophage polarization or neutrophil recruitment. Conversely, the adaptive immunity-dominant cluster A might benefit from immunomodulatory regimens such as B cell depletion or T cell checkpoint inhibition. Future studies should correlate these subtypes with clinical outcomes—including NIHSS scores, infarct volume, and long-term functional recovery—to validate their potential in guiding precision medicine for IS patients.

### Causal role of *ARG1* in IS and its cellular localization

Although transcriptomic analyses can reveal associations, they are unable to establish causal relationships due to potential confounding factors. To address this, we employed a two-sample MR approach using expression quantitative trait locus SNPs as instrumental variables, a method that reduces confounding and reverse causality through genetic randomization.^45^^,^^46^ After clumping SNPs in linkage disequilibrium (r^2^ < 0.001), we identified 24 eQTLs linked to the three core genes, with high average F-statistics (ranging from 21.30 to 1884.49) confirming the strength of the instrumental variables. MR results demonstrated a significant causal protective effect of *ARG1* on IS risk (IVW method; FDR-corrected *p* = 0.03), whereas *VIM* and *PRPF31* exhibited no significant causal association. Sensitivity analyses, including scatter, forest, funnel, and leave-one-out plots, further supported the causal role of *ARG1* and ruled out instrumental variable bias or pleiotropy.

To delineate the cellular origin of *Arg1* in stroke, we first analyzed single-cell RNA sequencing data from a mouse IS model, which identified specific upregulation of *Arg1* within macrophages. To translate and extend these findings to human pathology, we performed scRNA-seq analysis on PBMCs from IS patients. This revealed a remodeled immune landscape and highlighted a cell-type-specific upregulation of *ARG1* in three myeloid subsets in IS samples: plasmacytoid dendritic cells, activated type 2 conventional dendritic cells, and classical monocytes. Collectively, these cross-species and multi-tissue analyses not only consistently pinpoint myeloid immune cells—encompassing macrophages, specific dendritic cell subsets, and monocytes—as key sources of elevated *ARG1* in stroke (directly addressing the limitation of bulk RNA-seq by clarifying the cellular origin of *ARG1* elevation in human peripheral blood) but also align with prior literature^47^^,^^48^^,^^49^ documenting that myeloid cells (monocytes, DC subsets) mediate neuroinflammation in IS through brain infiltration and immune response regulation. Our data further extend this knowledge by linking these myeloid populations to *ARG1*-driven pathways.

Given the well-documented role of monocytes infiltrating the ischemic brain and differentiating into macrophages,^48^^,^^50^^,^^51^^,^^52^ it is plausible that these *ARG1*-high circulating monocytes contribute to the population of *ARG1*-expressing macrophages identified in the mouse MCAO model. These findings suggest that *ARG1* contributes to IS pathogenesis through myeloid cell-mediated mechanisms. These may include arginine depletion—impairing nitric oxide synthesis and vascular relaxation—and inhibition of T cell activation, thereby disrupting immune homeostasis and delaying tissue repair.^53^^,^^54^ The convergent evidence nominates *ARG1* as a promising therapeutic target.^55^ Specifically inhibiting *ARG1* within these pathogenic myeloid populations offers a potential strategy to mitigate ischemic injury while avoiding systemic immunosuppression.

Our study delineates the role of deacetylation-related genes in IS through integrated analyses. We identified 52 differentially expressed DEARGs enriched in pathways including protein acetylation, NF-κB signaling, and Th17 cell differentiation. A three-gene diagnostic signature (*VIM*, *ARG1*, and *PRPF31*) was constructed using machine learning and showed high accuracy (training *AUC* = 1.000; validation *AUC* = 0.840). This signature stratified IS patients into two molecular subtypes with distinct immune microenvironments. Mendelian randomization established *ARG1* as a causal factor for IS (IVW method, 11 SNPs, raw *p* = 0.004, FDR-corrected *p* = 0.03), and cross-species single-cell RNA-seq localized *ARG1* upregulation to myeloid cells—including brain macrophages, classical monocytes, and specific dendritic subsets. Collectively, our work defines a “deacetylation-immune” axis in IS pathogenesis, providing a foundation for *ARG1*-targeted diagnostics and therapies.

### Limitations of the study

This study has several limitations. The analyses primarily rely on retrospective public datasets, which may introduce sample heterogeneity. Although cross-species single-cell data (mouse and human) were integrated, species differences must be considered in translational interpretation. Despite strong genetic and transcriptomic evidence implicating *ARG1*, causal validation through cell-specific functional experiments is still lacking. Furthermore, the three-gene signature and molecular subtypes require validation in multi-center prospective studies to assess their clinical efficacy and prognostic value. The proposed “deacetylation-immune” axis is largely inferred from transcriptomic correlations; elucidating its underlying mechanisms will require direct measurements of enzymatic activity, protein modifications, and metabolic changes. Therefore, future studies should focus on: performing macrophage-specific functional validation of *ARG1*; advancing prospective clinical validation of the signature and subtypes; integrating multi-omics data to empirically define the regulatory network; and exploring their potential for patient stratification in targeted therapies.

## Resource availability

### Lead contact

Requests for further information and resources should be directed to and will be fulfilled by the lead contact, Wei Hu (huwei@hospital.westlake.edu.cn).

### Materials availability

This study did not generate new unique reagents.

### Data and code availability

• This paper analyzes existing, publicly available data, accessible at GEO: GSE16561, GSE267240, GSE37587, GSE22255, GSE285659, GSE199819, and GSE58294.
• All codes were used in this study in alignment with recommendations made by authors of R packages in their respective user’s guide, which can be accessed at https://bioconductor.org.
• Any additional information required to reanalyze the data reported in this paper is available from the lead contact upon request.

## Acknowledgments

This research was actually supported by the Hangzhou Joint Fund of Zhejiang Provincial Natural Science Foundation of China (grant no. LHZQN25H160001); the medical and health research project of Zhejiang Province (grant no. 2025KY118); the Construction Fund of Key Medical Disciplines of Hangzhou (grant: 2025HZZD04); and Medical and Health Technology Project of Hangzhou (grant: Z20240021).

## Author contributions

Conceptualization, H.-Z.R. and Y.Z.; methodology, H.-Z.R., S.-C.C., and Y.Z.; investigation, H.-Z.R., Y.Z., S.-C.C., J.-Y.H., and W.H.; writing—original draft, H.-Z.R.; writing—review & editing, H.-Z.R. and W.H.; funding acquisition, H.-Z.R. and W.H.; supervision, J.-Y.H and W.H. All authors interpreted the results and read and approved the final version of the manuscript.

## Declaration of interests

The authors declare no competing interests.

## Declaration of generative AI and AI-assisted technologies in the writing process

No generative AI or AI-assisted technologies were used in the writing or preparation of this manuscript.

## STAR★Methods

### Key resources table

**Table:** 

REAGENT or RESOURCE	SOURCE	IDENTIFIER
**Chemicals, peptides, and recombinant proteins**		

RNasin® Plus Ribonuclease Inhibitor	Promega	Catalog Number: N2611

**Critical commercial assays**		

Triquick Reagent Kit	Solarbio Life Sciences	Catalog Number: R1100
PrimeScript™ RT Master Mix	TaKaRa	Catalog Number: RR036A
QuantiNova Probe PCR Kit	QIAGEN	Catalog Number: 208252

**Deposited data**		

Transcriptome data from IS patients	GEO	GSE58294
Transcriptome data from IS patients	GEO	GSE22255
Transcriptome data from IS patients	GEO	GSE16561
Transcriptome data from IS patients	GEO	GSE37587
Processed single-cell RNA- seq data	GEO	GSE285659
Transcriptome data from IS patients	GEO	GSE199819
Processed single-cell RNA- seq data	GEO	GSE267240
The eQTL data	the eQTLGen Consortium	https://eqtlgen.org/
The gene signatures of 22 immune cell types	CIBERSORT	https://cibersort.stanford.edu/
The IS cohort dataset	FinnGen R12	https://r12.finngen.fi/

**Experimental models: organisms/strains**		

Sprague-Dawley rats	Hangsi Biological Technology	N/A

**Oligonucleotides**		

Primer *Vim* F: GACGCCATCAACACCGAGT	This paper	Sangon Biotech
Primer *Vim* R: CATTTCACGTCGTCGCGGAA	This paper	Sangon Biotech
Primer *Arg1* F: TGCTGAAGGACACCTTTGCT	This paper	Sangon Biotech
Primer *Arg1* R: GGAGGTCTTGGTGATGTCCT	This paper	Sangon Biotech
Primer *Prpf31* F: AGAACCCAGAGCCAGAAGGA	This paper	Sangon Biotech
Primer *Prpf31* R: TCCACGATGGTCTTGATGGT	This paper	Sangon Biotech
Primer *Gapdh* F: GGGTGTGAACCATGAGAAGT	This paper	Sangon Biotech
Primer *Gapdh* R: CAGTGATGGCATGGACTGTG	This paper	Sangon Biotech

**Software and algorithms**		

RStudio software version 4.5.2	The R Project	RRID:SCR_000432
VennDiagram package	https://www.r-project.org	v.1.7.3
sva package	https://www.r-project.org	v.3.8.3
limma package	https://www.r-project.org	v.3.60.6
ComplexHeatmap package	https://www.r-project.org	v.2.26.0
ggplot2 package	https://www.r-project.org	v.3.5.1
e1071 package	https://www.r-project.org	v.1.7-14
randomForest package	https://www.r-project.org	v.4.7-1.1
glmnet package	https://www.r-project.org	v.4.1-8
xgboost package	https://www.r-project.org	v.3.1.2.1
pROC package	https://www.r-project.org	v.1.18.5
GSVA package	https://www.r-project.org	v.2.2.0
ConsensusClusterPlus	https://www.r-project.org	v.1.16.0
Seurat package	https://www.r-project.org	v.5.3.1
clusterProfiler package	https://www.r-project.org	v.4.12.0
MendelianRandomization	https://www.r-project.org	v.0.10.0
MR-PRESSO	https://www.r-project.org	v.1.0
TwoSampleMR packages	https://mrcieu.github.io/TwoSampleMR/	v.0.6.15
Adobe Illustrator 2020 version 24.0.1	Adobe Inc.	RRID:SCR_010279

### Experimental model and study participant details

#### Animals

Male Sprague-Dawley (SD) rats of wild-type genotype, weighing 180–220 g and aged approximately 8–10 weeks (young adult stage), were obtained from Hangsi Biological Technology Co., Ltd. (Hangzhou, China), a certified commercial supplier. Upon arrival, animals were acclimatized to the laboratory environment for 3–5 days to minimize stress-related confounding effects. They were housed under standard conditions: two rats per cage with sterile bedding renewed every 2 days, controlled temperature (25 ± 1 °C), relative humidity (50 ± 5%), and a 12 h light/dark cycle (lights on 08:00–20:00). All rats had free access to standard rodent chow and sterile drinking water throughout the study, and daily health monitoring was performed to exclude any signs of illness or abnormal behavior. All experimental procedures were conducted in accordance with the guidelines for the care and use of laboratory animals and were approved by the Laboratory Animal Experimental Ethical Inspection Committee of Dr.Can Biotechnology (Zhejiang) Co., Ltd. (DRK2024022201). Only male rats were used, and the potential influence of sex on cerebral ischemia-reperfusion injury and related outcomes is acknowledged as a limitation, as female rats were not included to assess sex-specific differences.

#### Middle cerebral artery occlusion (MCAO)

Focal cerebral ischemia was induced in rats by transient MCAO following the Longa method.^56^ Under pentobarbital anesthesia (40 mg/kg, i.p.), a cervical incision was made to expose the carotid arteries. A silicone-coated monofilament was inserted via the external carotid artery and advanced to occlude the MCA for 2 hours, followed by reperfusion. Sham-operated controls received identical surgery without filament insertion.

### Method details

#### Data acquisition

Gene expression data were acquired from the Gene Expression Omnibus (GEO) database (http://www.ncbi.nlm.nih.gov/geo/) under accession numbers GSE16561, GSE267240, GSE37587, GSE22255, GSE285659, GSE199819, and GSE58294. The datasets GSE22255 and GSE58294 (platform: GPL570) were merged to form a combined cohort consisting of 89 IS patients and 43 healthy controls, with batch effects adjusted using the ComBat algorithm from the sva R package.^57^ Similarly, GSE16561 and GSE37587 (platform: GPL6883) were integrated into an independent validation cohort—with batch effects adjusted using the same algorithm—comprising a final set of 88 IS samples and their corresponding 24 healthy controls. Genes related to deacetylation were identified from the GeneCards database (https://www.genecards.org/) using the keyword “deacetylation”. Those with a relevance score cutoff of ≥0.156 to ensure high-confidence associations. Additionally, we filtered for genes with a GeneCards Inferred Functionality Score (GIFtS) > 2. This multi-step filtering process resulted in a final list of 3,158 genes.^58^ The overlap between differentially expressed genes (DEGs) in IS and the deacetylation-related genes was visualized using the VennDiagram R package.

#### Inclusion and exclusion criteria for the validation dataset

Inclusion criteria: (1) Samples were clearly annotated as “IS patients” or “healthy controls” with definite clinical diagnostic criteria (consistent with the WHO definition of IS); (2) Complete gene expression data were available, including the three core signature genes (*VIM, ARG1, PRPF31*) without missing values;(3) No obvious experimental artifacts were present (e.g., extreme outlier values in gene expression intensity, as detected via boxplot and principal component analysis [PCA] preprocessing). Exclusion criteria: (1) Samples with ambiguous diagnostic labels (e.g., “suspected IS” or comorbid cerebrovascular disease); (2) Samples lacking key gene expression data (i.e., missing values for ≥1 of the three core genes after data normalization); (3) Outlier samples for which batch effects could not be corrected (identified via PCA, where samples deviating significantly from the main cluster of their respective groups were excluded); (4) Duplicate samples or samples from the same patient (confirmed via sample metadata and clinical information).

#### Differentially Expressed Genes (DEGs) analysis between stroke patients and healthy controls

DEGs between stroke patients and healthy controls were identified with the “limma” R package, with significance defined as an absolute log_2_-fold change ≥1 and an FDR-adjusted *p* ≤ 0.05.^59^ Co-expression relationships among deacetylation-related genes were analyzed via correlation, with Pearson’s or Spearman’s rank correlation selected based on rigorous pre-verification of normality and linearity assumptions for gene expression distributions. The Shapiro-Wilk test (*p* > 0.05) was used to assess expression normality for each gene; only passing gene pairs underwent linearity verification via residual analysis combined with the Durbin-Watson test (statistic: 1.5–2.5). Pearson’s correlation was applied to gene pairs meeting both assumptions, while Spearman’s rank correlation was used for those violating either. All normality/linearity verification results and corresponding visualizations are provided in the supplementary materials; the Benjamini-Hochberg (BH) multiple testing correction was applied to all correlation results, with statistical significance set at FDR-adjusted *p* < 0.05. These DEGs were visualized by volcano plot and a heatmap was constructed using the R packages ComplexHeatmap and ggplot2 (version 3.5.1).

#### Functional analysis of DEGs

To elucidate the biological functions and pathways associated with the identified DEGs, Gene Ontology (GO), Disease Ontology (DO) and Kyoto Encyclopedia of Genes and Genomes (KEGG) enrichment analyses were performed using the clusterProfiler R package to identify statistically over-represented terms and to explore the possible mechanism(s) by which DEGs participate in IS.^60^^,^^61^

#### Machine learning algorithms

To identify hub genes for IS diagnosis, we applied four machine learning algorithms: Extreme Gradient Boosting (XGBoost),^62^ Support Vector Machine-Recursive Feature Elimination (SVM-RFE),^63^ Random Forest (RF),^64^ and Least Absolute Shrinkage and Selection Operator (LASSO). These four algorithms were selected by aligning with our research objective (screening robust hub genes for the IS diagnostic signature) and the inherent characteristics of our transcriptomic data (high-dimensional gene expression profiles, relatively small sample size, and potential non-linear gene-IS associations), given their unique and complementary strengths in feature selection and predictive modeling: (1) LASSO was selected for its robust L1 regularization, which efficiently compresses redundant gene features to zero and identifies core genes with the most significant diagnostic value for IS, thus making it highly suitable for high-dimensional omics data; (2) SVM-RFE was selected for its recursive feature elimination mechanism that iteratively removes the least important features and optimizes the classification hyperplane, and it is well-adapted to the small-sample and high-dimensional properties of our gene expression dataset, stably identifying gene subsets with strong discriminatory power between IS and healthy control samples; (3) RF was selected for its superior ability to handle non-linear and interactive gene relationships—a common property of transcriptomic data—while quantifying feature importance and mitigating overfitting via bootstrap sampling and 10-fold cross-validation; (4) XGBoost, an integrated learning algorithm, was adopted to further improve the predictive accuracy of the diagnostic model: it integrates the strengths of multiple base learners, iteratively corrects prediction errors, and tolerates minor noise in gene expression data, thereby enhancing the reliability of hub gene screening. The SVM-RFE analysis was implemented with the e1071 package, where hub genes served as independent variables for constructing an optimal hyperplane that maximized the inter-class margin. The RF analysis was performed using the randomForest package with 10-fold cross-validation. LASSO regression was implemented with the glmnet package via 5-fold cross-validation (lambda.1se as the optimal penalty parameter) to perform feature shrinkage and selection. XGB analysis was conducted with the xgboost package, where key hyperparameters (learning rate = 0.1, max_depth = 3, nrounds = 100) were optimized via grid search. The predictive performance of all four models was evaluated by receiver operating characteristic (ROC) analysis using the pROC package, and the area under the curve (AUC) was calculated to quantify classification accuracy.^65^

#### Estimating immune cell infiltration

To characterize the immune infiltration landscape in IS, we employed two computational approaches. First, the CIBERSORT algorithm was used to deconvolute the bulk transcriptomic data and estimate the relative abundances of 22 immune cell types based on their specific gene signatures (https://cibersort.stanford.edu/).^66^ Subsequently, differences in the enrichment fractions of distinct immune cell subsets were compared via the Wilcoxon rank-sum test. Additionally, quantitative single-sample gene set enrichment analysis (ssGSEA) was performed to quantify the relative abundance of immune cells associated with IS-protein deacetylation interactions. The immune cell-related gene sets used in ssGSEA were primarily derived from the Molecular Signatures Database (MSigDB, v7.5.1, Subcollection C7: Immunologic Signatures), as referenced in Wang, Y. et al.,^67^ which curates experimentally validated gene sets for immune cell subsets. Finally, variations in immune cell infiltration patterns were visualized using the ggplot2, GSVA packages in R.^68^^,^^69^

#### Unsupervised clustering analysis

To identify molecular subtypes based on DEARG expression, we performed unsupervised consensus clustering on the stroke patient samples using the ConsensusClusterPlus R package.^70^ This analysis categorized the samples into two distinct deacetylation clusters. Consensus clustering was conducted with the following parameters, selected to match the characteristics of our IS-related deacetylation gene expression data and adhere to clustering technical standards: maximum cluster number (maxK) = 9: to comprehensively evaluate the optimal cluster number within a rational range, preventing subtype omission (unduly low maxK) and biologically meaningless over-clustering (unduly high maxK), in line with classic ConsensusClusterPlus guidelines for transcriptomic data; proportion of items sampled (pItem) = 0.8: a universally accepted ratio in consensus clustering that balances sample representativeness and randomness, verifying clustering robustness to sampling variation; proportion of features sampled (pFeature) = 1: to retain all pre-screened IS-relevant deacetylation-related genes, avoiding the loss of key molecular signals associated with deacetylation and IS heterogeneity; hierarchical clustering (hc) with Spearman distance as the metric: this metric is robust to non-normal distributions and outliers in IS transcriptomic data, and outperforms Euclidean distance in quantifying inter-sample gene expression profile similarity; 1,000 iterations (a consensus clustering standard): to minimize random sampling bias, confirm the stability of the two identified deacetylation clusters, and ensure experimental reproducibility. Principal component analysis (PCA) was then used to visualize cluster segregation. Furthermore, Spearman correlation analysis was applied to assess the relationships between the expression of key deacetylation-related genes and immune cell infiltration levels.^71^

#### Calculation of deacetylation score

The PCA algorithm was used to calculate the deacetylation score of each sample to quantify deacetylation patterns. The deacetylation score was calculated as Σ (Expi ∗ PCi), where PCi and Expi represent the principal components and expression level of each gene, respectively.^72^ The cutoff for classifying samples into high- and low-score groups was the median value of the deacetylation scores across all IS samples.

#### Summary data-based mendelian randomization analysis

We applied a two-sample MR approach to evaluate the causal effects of key DEG profiles (exposure) on embolic IS (outcome). As described in previous studies,^73^^,^^74^ genetic variants (single nucleotide polymorphisms, SNPs) of key DEGs identified via machine learning algorithms were selected as instrumental variables (IVs). SNPs serving as IVs for each key DEG were extracted from the Integrative Epidemiology Unit (IEU) Open GWAS database (https://gwas.mrcieu.ac.uk/), specifically from whole-blood eQTL datasets of European (EUR) ancestry. A strict genome-wide significance threshold (*p* < 5 × 10^−8^) was set to ensure robust associations between SNPs and target DEG expression. Additional SNP quality control included excluding SNPs located in the HLA region, duplicate variants, and insertion/deletion polymorphisms. Remaining SNPs were clumped to retain independent genetic variants using PLINK software, with parameters set as linkage disequilibrium (LD) r^2^ < 0.001, clumping distance = 1,000 kb, and the 1,000 Genomes Project EUR panel as the LD reference. To mitigate weak instrument bias, IVs with an *F*-statistic >10 (calculated via linear regression of SNP genotypes on DEG expression levels) were retained.^73^^,^^74^ All datasets (eQTL and outcome) were restricted to individuals of EUR ancestry to minimize population stratification bias. IS outcome data were retrieved from the FinnGen database (R12 release, https://r12.finngen.fi/), with the specific phenotype defined as “embolic IS”. This dataset included 1,808 cases and 453,828 controls, with a total sample size of 455,636.

#### Sensitivity analysis

The strength of SNPs as IVs was assessed using the F-statistic, with only those exhibiting an F-statistic >10 included to minimize weak instrument bias.^73^ Additionally, the Heterogeneity in Independent Instruments (HEIDI) test was performed via SMR v1.3.1 to distinguish true pleiotropy from linkage-driven associations: all IVs with a HEIDI *p*-value <0.01 (a predefined threshold indicating significant heterogeneity suggestive of linkage effects rather than target gene-mediated associations) were excluded from the analysis. For sensitivity analyses, we compared causal estimates across multiple MR methods—including MR-Egger, penalized weighted median MR, simple mode, random-effects IVW, and weighted mode—to enhance the robustness of our findings. We utilized forest plots to assess the causal effects of individual SNPs and compare them against estimates derived from IVW and MR-Egger (employing all enrolled SNPs). Potential directional pleiotropy was examined by evaluating funnel plot asymmetry, combined with the MR-Egger intercept test to statistically validate the significance of asymmetry, thereby gauging the reliability of the MR analyses.

#### The scRNA-seq data processing

The 10x scRNA-seq data from datasets GSE267240 and GSE285659 were processed and analyzed using the Seurat package (v5.3.1). Following acquisition, quality control was performed by filtering out genes expressed in fewer than 3 cells and cells containing fewer than 200 genes. Cells were further filtered based on the following QC thresholds: nFeature_RNA between 500 and 4,000, nCount_RNA below 30,000, and percent.mt below 20%. The data were then normalized, and the top 2,000 highly variable genes were identified for scaling and principal component analysis. Significant principal components were selected using the Jackstraw procedure (*p* < 0.05) for subsequent unsupervised clustering (FindNeighbors and FindClusters functions, resolution = 0.1). Cell clusters were visualized using UMAP and annotated into distinct cell types based on established marker genes reported in the literature.^75^

#### Reverse transcription-quantitative PCR (RT-qPCR)

Total RNA was isolated from frozen MCAO and normal tissue samples using the Triquick reagent kit (Solarbio Life Sciences). Complementary DNA (cDNA) was synthesized from the extracted RNA using PrimeScript RT Master Mix (Takara). Primer sequences for target genes are provided in Table S2. GAPDH served as the reference gene, and the 2ˆ(-ΔΔCt) method was applied to calculate relative expression. Data were analyzed by the Mann-Whitney U test, with *p* < 0.05 considered significant, and visualized using R software.

#### Statistical analysis

All statistical analyses were conducted using RStudio 4.5.2. Continuous variables between two independent groups were compared using the non-parametric Wilcoxon rank-sum test, as the data did not meet the assumptions of normality and homogeneity of variances for the parametric independent samples *t* test. For comparisons of continuous variables across three or more independent groups that did not meet the assumptions of normality and homogeneity of variances, the Kruskal–Wallis test was performed to assess the overall group differences. Only when a statistically significant overall difference was observed, post-hoc pairwise comparisons were conducted using Dunn’s test, and the raw *p*-values from Dunn’s test were further adjusted with the Bonferroni correction to control the family-wise error rate (FWER). Categorical variables were analyzed via the chi-square test (large sample sizes with expected frequencies ≥ 5) or the Fisher’s exact test (small sample sizes with expected frequencies < 5). The Benjamini-Hochberg (BH) false discovery rate correction method was applied to the raw *p*-values of all pairwise correlation analyses (both Pearson’s and Spearman’s) to control FDR, with statistical significance for correlations defined as an FDR-adjusted *p* < 0.05; all BH-corrected results are provided in the supplementary materials.

We used a two-sample MR approach to evaluate the causal effects of three key independent exposure genes on IS incidence, with results presented as ORs with 95% CIs. For statistical rigor in multiple independent MR analyses, two complementary multiple testing correction strategies—standard for small-scale MR research—were used: Bonferroni correction (controls FWER, threshold: 0.05/3 ≈ 0.0167) and Benjamini-Hochberg FDR correction (threshold: 0.05). All corrected P-values were calculated via R’s p.adjust() function. To address potential heterogeneity and horizontal pleiotropy, five MR methods were used: random-effects IVW,^76^ weighted median,^77^ MR-Egger,^78^ simple mode,^79^ and weighted mode. Additionally, the MR-PRESSO test detected and calibrated pleiotropic outliers in summary-level analyses, while heterogeneity among genetic variant-derived causal estimates was assessed via scatterplots and Cochran’s Q tests.^80^ All two-sample MR analyses were performed with the MendelianRandomization, MR-PRESSO, and TwoSampleMR R packages (v0.6.15, Hemani et al.), publicly available at GitHub (https://mrcieu.github.io/TwoSampleMR/).^79^

### Quantification and statistical analysis

All statistical details are described in the figure legends and in the STAR Methods.
