# Supplementary material for: Integrated multi-omics identifies macrophage ARG1-mediated deacetylation with causal and diagnostic implications in ischemic stroke
Source: iScience. 2026 Mar 6;29(4):115269. doi: 10.1016/j.isci.2026.115269 (PMC13018907; doi:10.1016/j.isci.2026.115269)
Supplement: Document S1. Figures S1–S5 [file mmc1.pdf]

iScience, Volume *29*

Supplemental information

Integrated multi-omics identifies macrophage ARG1-mediated deacetylation with causal and diagnostic implications in ischemic stroke

Hang-Ze Ruan, Ying Zhu, Shen-chi Cheng, Jin-Yu Huang, and Wei Hu

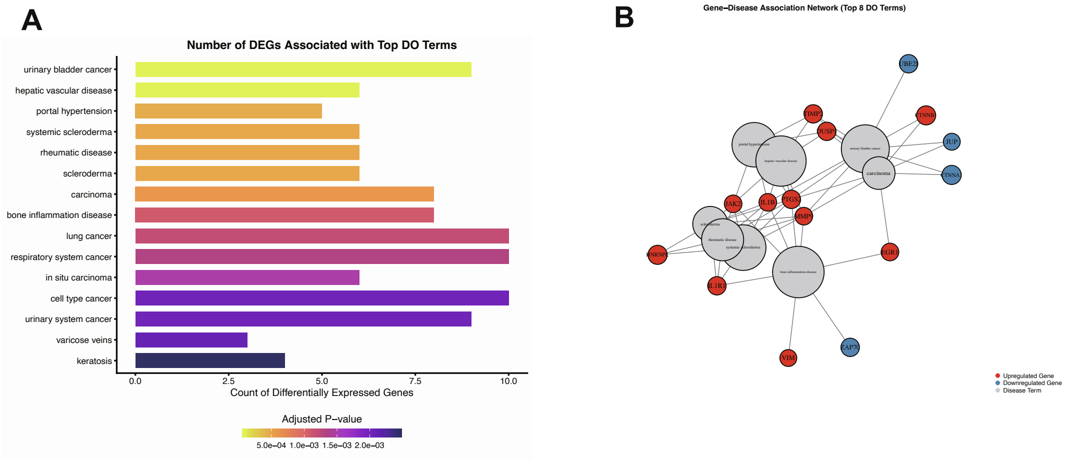

Figure S1. Disease Ontology (DO) enrichment analysis of differentially expressed genes. (A) Results of the DO enrichment analysis. Bar plot showing the number of DEGs associated with top-ranked DO terms (each term corresponds to a distinct disease). (B) Gene-disease association network for the top 8 DO terms, which visualizes the specific connections between DEGs, their corresponding diseases, and the statistical significance of these associations.

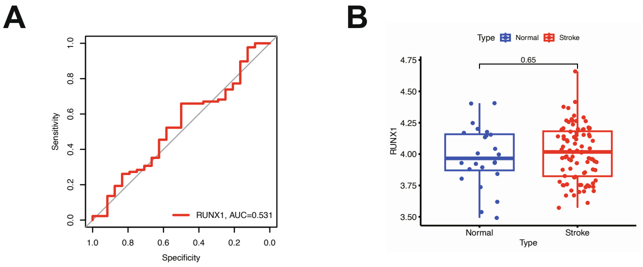

Figure S2. Validation of the diagnostic value of the *RUNX1* signature. (A) ROC curves showing the diagnostic value of the *RUNX1* signature in the GSE16561-37587 dataset. (B)The expression levels of *RUNX1* in normal and IS samples were validated in the GSE16561-37587 dataset.

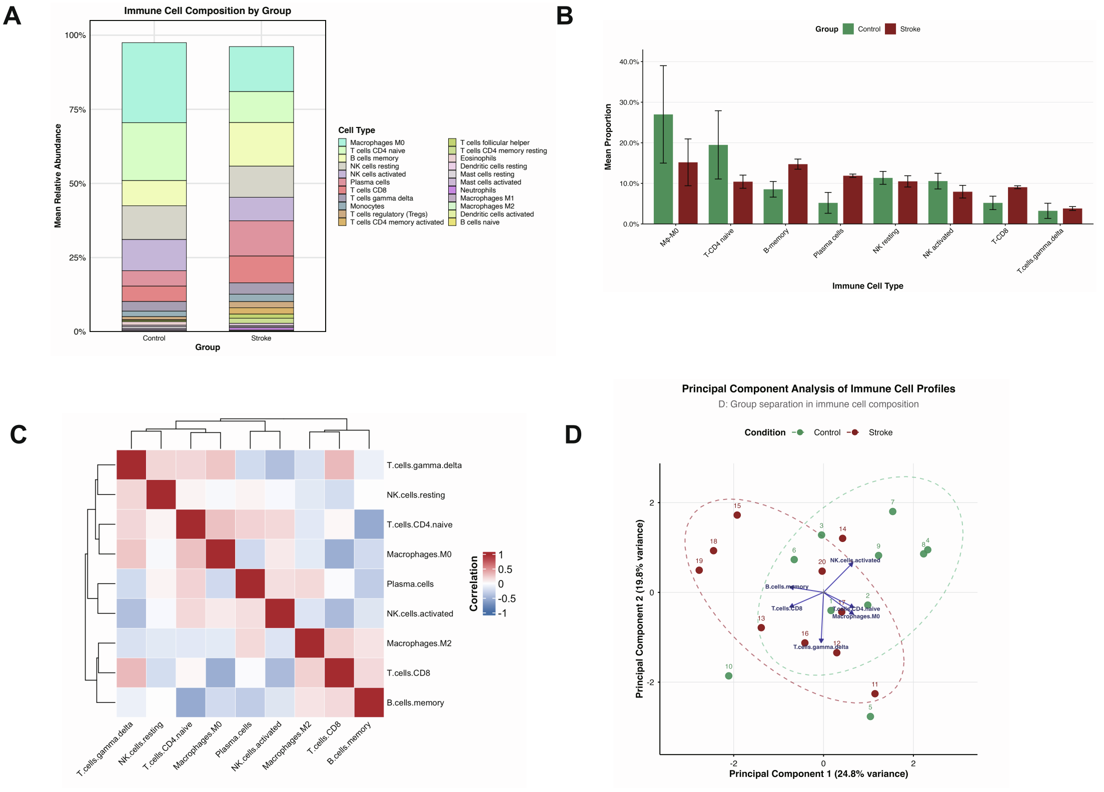

Figure S3. Profiling of PBMCs using CIBERSORT across all samples. (A) Immune cell infiltration analysis of the GSE199819 dataset was performed using the CIBERSORT algorithm, showing the composition of 22 immune cell types in IS and normal samples. (B) Comparison of the proportions of 8 immune cell types among different samples. (C) Correlation heatmap of pairwise Pearson correlation coefficients for immune cell subtype abundances. (D) Principal component analysis (PCA) plot of immune cell profiles (each point = 1 sample; color-coded: green = control, maroon = stroke). Generated from all subtype abundances; PC1 explains 24.1% of the variance, and PC2 explains 13.8%. Dashed 95% confidence ellipses indicate group clustering, demonstrating the separation of control and stroke samples based on immune cell composition.

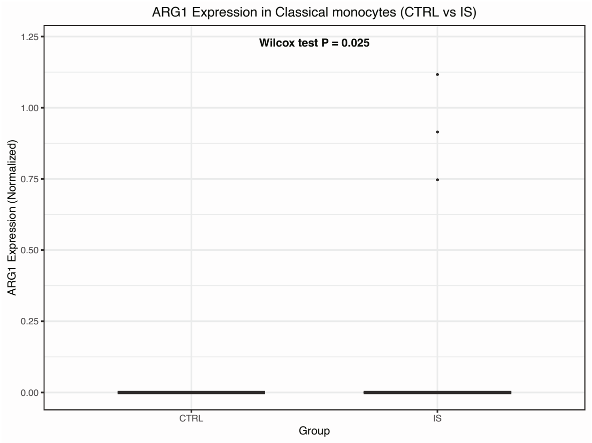

Figure S4. Transcriptomic expression profiling of *ARG1* in classical monocytes: comparative analysis between healthy controls and IS samples. This plot demonstrates normalized *ARG1* expression levels is statistically significantly elevated in the IS samples cohort relative to the healthy control. Wilcoxon rank-sum test yielding a *p*-value of 0.025.

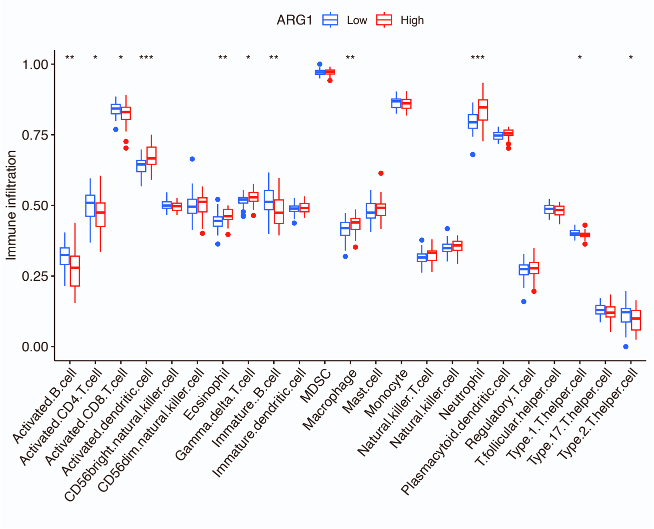

Figure S5. Difference in the abundance of infiltrating immune cells with different expression levels of *ARG1*. *$p < 0.05$; **$p < 0.01$; ***$p < 0.001$ by Wilcoxon rank-sum test.
